# Advances in Aptamer Diagnostics Targeting Non-Small-Cell Lung Cancer: A Systematic Review

**DOI:** 10.3390/ijms27156957

**Published:** 2026-08-03

**Authors:** Lisa Agnello, Ilaria Leone, Alessandra Affinito, Carla Lucia Esposito, Silvia Catuogno, Anna D’Agostino, Marco Salvatore, Gerolama Condorelli, Silvia Nuzzo

**Affiliations:** 1 IRCCS SYNLAB SDN, 80146 Naples, Italy; ilaria.leone@synlab.it (I.L.); alessandra.affinito@synlab.it (A.A.); marco.salvatore@synlab.it (M.S.); nuzzo.silvia@gmail.com (S.N.); 2 Institute of Endotypes in Oncology, Metabolism and Immunology “G. Salvatore” (IEOMI), Consiglio Nazionale delle Ricerche (CNR), 80146 Naples, Italy; c.esposito@cnr.ieos.it (C.L.E.); s.catuogno@ieos.cnr.it (S.C.); gecondor@unina.it (G.C.); 3 Department of Molecular Medicine and Medical Biotechnology, “Federico II” University of Naples, 80146 Naples, Italy

**Keywords:** aptamer, diagnostic, NSCLC, liquid biopsy, detection, fluorescence, exosome, CTC, protein

## Abstract

Non-small-cell lung cancer (NSCLC) is one of the most frequent cancer types and is responsible for the majority of cancer-related deaths worldwide. For this reason, initial diagnosis, prognosis, and targeted therapy of NSCLC represent very attractive areas of study. Aptamers are single-stranded nucleic acids (RNA or DNA) generated through Systematic Evolution of Ligands by Exponential Enrichment (SELEX); they are able to bind to a molecular target with high affinity and specificity. Thanks to their intrinsic nucleic acid properties, they can be easily modified and optimized to enhance target binding and their half-life; moreover, they exhibit no immunogenicity and toxicity. Due to this, many aptamers have just been selected against NSCLC biomarkers and provide specific imaging agents to improve the diagnosis of this type of cancer. However, despite the promising results in preclinical studies, the application of aptamers in NSCLC diagnosis is still in its early stages, mainly due to the limited literature in the research world, the dominance of antibodies in the pharmaceutical market and the challenge of target identification. Consequently, this appears to be a temporary issue, and aptamers could see increasing application in the future; thus, we performed a review aimed at summarizing current knowledge on the new promising DNA and RNA aptamer-based molecules for NSCLC diagnosis. All studies from 2000 were included and investigated. Our findings showed that several DNA and RNA aptamers are promising diagnostic tools for NSCLC management.

## 1. Introduction

Cancer detection provides a wide range of strategies designed to identify tumor cells or neoplastic lesions at an early stage. Over the past decades, diagnostic approaches have been switched from simple morphological observations to advanced live imaging techniques.

In this specific case, non-small cell lung cancer (NSCLC) represents the most common cause of cancer-related deaths [1] and is diagnosed at an advanced stage [2] through a process that integrates several clinical, radiological, and laboratory steps. In most cases, initial suspicion arises from the presence of symptoms that lead to further investigations such as techniques based on computed tomography (CT), useful for clarifying the extent of the disease.

A definitive diagnosis, however, requires histological confirmation, obtained through tumor tissue sampling [3].

In addition to histological diagnosis, molecular characterization is essential. Indeed, the major obstacle to early and accurate NSCLC diagnosis is the marked biological heterogeneity that characterizes the disease at multiple levels. Inter-patient heterogeneity is evident in the distribution of driver oncogenic alterations. Specific markers, such as TTF-1 or p40, confirm the tumor type, while molecular analyses allow for the identification of relevant genetic alterations, such as EGFR mutations, ALK and ROS1 rearrangements, KRAS or BRAF mutations, and other alterations in MET, RET, or NTRK. Today, routine molecular tests for identifying EGFR mutations and ALK rearrangements have changed the therapeutic management of NSCLC, allowing the use of highly effective tyrosine kinase inhibitors [4]. It is important to note that both ALK and ROS1 are screened by immunohistochemistry, but positive cases require FISH (or Next Generation Sequencing) confirmation to avoid false positives due to non-specific staining [4].

However, the interpretation of molecular findings remains challenging due to intratumoral heterogeneity. In fact, intra-tumoral difference further complicates diagnosis, and molecular profiles dynamically evolve under therapeutic pressure, and thus a single biopsy site may not represent the complete molecular profile of the tumor. This heterogeneity extends to protein expression: PD-L1, for instance, can vary substantially between the primary tumor and metastatic sites, and even across different regions of the same lesion [5], and it also evolves temporally under the selective pressure of therapy, as exemplified by the emergence of the secondary EGFR mutations (T790M) resistance mutation [6], MET amplification following tyrosine kinase inhibitor treatment [7] or ALK resistance mutations [8], histological transformation, and several other mechanisms of adaptive resistance. Importantly, NSCLC is a highly dynamic disease characterized by continuous clonal evolution and repeated changes in molecular profiles, making the process of diagnosing and monitoring the disease complex. Therefore, these layers of heterogeneity mean that diagnostic strategies relying on a single biomarker or a single sampling time-point are inherently limited, and they provide a strong rationale for multiplexed, non-invasive approaches.

Prognosis is strongly stage-dependent: five-year survival exceeds 60% for localized disease but falls below 10% once distant metastases are present, which is the case in most patients at diagnosis. This steep survival gradient is the principal rationale for developing sensitive, minimally invasive diagnostic tools capable of detecting disease at an earlier, more treatable stage.

Luckily, over the past decades, many novel studies are looking at making diagnostic techniques more precise and less invasive, even for NSCLC, and once again aptamers appear as a useful tool; indeed, combining the sensitivity, selectivity, and real-time visualization of tumor biomarkers, aptamers are gaining increasing attention as innovative probes for diagnostic imaging. Indeed, to this aim, nucleic acid aptamers show interesting properties that position aptamers as a chemically defined alternative to monoclonal antibodies, the recognition elements that currently dominate clinical diagnostics; a detailed side-by-side comparison of the two classes is provided in Table 1.

Aptamers were first described in 1990 through two independent in vitro selection strategies [9,10], SELEX and in vitro evolution, and have since been generated against an extensive range of targets, from small metabolites to whole cells.

Unlike antibodies, whose specificity arises from hypervariable protein loops, aptamers achieve target recognition through sequence-directed folding of a single-stranded oligonucleotide into a defined three-dimensional architecture, such as hairpins, stem-loops, pseudoknots or G-quadruplexes; the resulting molecular surface engages the target through a combination of hydrogen bonding, electrostatic complementarity, base stacking and shape complementarity.

Their dissociation constants (Kd) typically fall in the nanomolar-to-picomolar range, comparable to or better than many monoclonal antibodies [11,12]. Unlike antibodies, which require the immunization of a living host or a cultured cell line, aptamers are entirely chemically synthesized, a feature that eliminates lot-to-lot biological variability and shortens development timelines from months to weeks. Their oligonucleotide backbone can be further stabilized against nuclease degradation through chemical modifications such as 2′-fluoro, 2′-O-methyl or locked nucleic acid substitutions, or using mirror-image L-nucleotides (Spiegelmers), extending their in vivo half-life without compromising folding or target recognition. Structured as single-stranded oligonucleotides, they can be internalized into cells and are easily engineerable and functionalized with fluorophores, radionuclides, or nanoparticles, making them particularly suitable for cutting-edge imaging modalities [13].

Nanoparticle internalization by tumor cells commonly relies on passive mechanisms, such as diffusion and the enhanced permeability and retention (EPR) effect, which exploit the leaky vasculature and poor lymphatic drainage typical of solid tumors but afford limited cell-type specificity. Conjugating aptamers to the nanocarrier surface convert this passive uptake into an active, receptor-mediated process, in which aptamer-target engagement triggers endocytosis specifically in cells expressing the cognate biomarker, thereby improving cellular uptake efficiency and reducing off-target accumulation. In particular, nanocarriers can be physically and stably conjugated to the aptamer itself, and the chemistry available for this coupling differs depending on whether the carrier is metallic or non-metallic. Metallic nanostructures, such as gold nanoparticles and gold electrodes, are most functionalized through direct thiol–gold (Au–S) covalent bonding, exploiting a terminal thiol group introduced during aptamer synthesis. Radiometal-based platforms instead require a bifunctional chelator, such as NOTA or DOTA, covalently attached to the aptamer to coordinate the metal ion (e.g., gallium-68) with high thermodynamic stability. Non-metallic carriers rely on a broader repertoire of bioconjugation strategies: amine- or carboxyl-terminated aptamers can be coupled to polymeric or silica nanoparticles via carbodiimide (EDC/NHS) chemistry; biotinylated aptamers exploit the biotin–streptavidin interaction for modular assembly on nanoparticle or membrane surfaces; and azide- or alkyne-functionalized aptamers can be conjugated through copper-catalyzed or strain-promoted click chemistry, offering high specificity and bio-orthogonality. The choice of functional group and conjugation chemistry therefore directly determines the stability, orientation, and functional accessibility of the aptamer once immobilized on its nanocarrier. Moreover, aptamers can be employed as stand-alone diagnostic probes, directly conjugated to a single reporter moiety such as a fluorophore, radiolabel, or chelated radiometal, without the need for a nanoparticle scaffold; this simpler format offers faster renal clearance.

These combined properties, low production variability, high affinity, chemical tunability and ease of functionalization, underline the growing interest in aptamers as an alternative or complementary class of recognition elements to antibodies in oncological diagnostics.

In this review, we will discuss various studies from 2005 to the present that address the use of aptamers for diagnostic purposes.

## 2. Materials and Methods

### 2.1. Search Strategy and Selection Criteria

**Figure 1:**
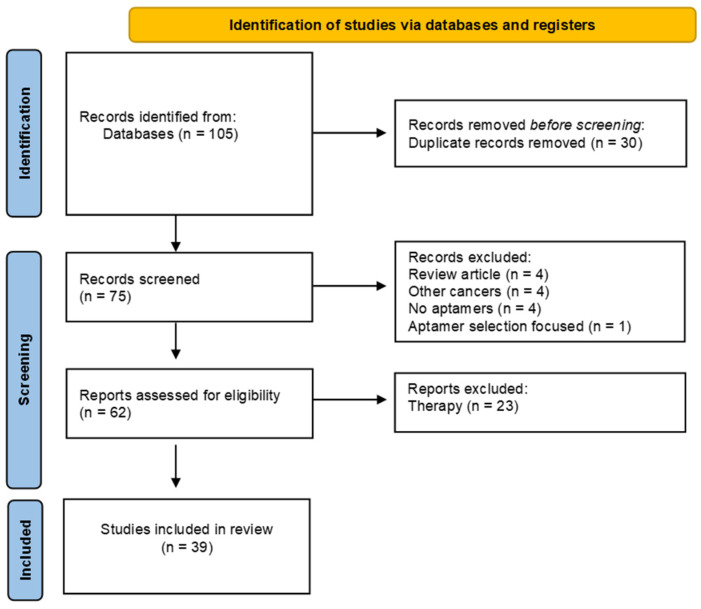
PRISMA 2020 flow chart of the literature search and study selection process for the systematic review. The PRISMA flow diagram of included studies according to the inclusion and exclusion criteria is reported in Figure 1, and their characteristics are summarized in Results paragraph.

This systematic review adheres to the PRISMA 2020 guidelines, as shown in the corresponding flow diagram (Prisma). A systematic literature search was conducted in PubMed (last search: October 2025) to identify all published studies concerning the application of aptamers as diagnostics for NSCLC. Five concept-specific search strings were used, combining the terms “aptamer”/“aptamers” and “non-small cell lung cancer” with an additional term restricting results to “diagnosis”, “capture”, “imaging”, “liquid biopsy”, or “sensor”, respectively (full search strings and per-query results reported in Supplementary Table S1 and Files S1a–S1e). Google Scholar, Web of Science, and Science Direct were additionally consulted using the same terms as a coverage check; these searches did not identify any eligible record beyond those already retrieved from PubMed, and the PRISMA flow reported in Figure 1 is therefore based on the PubMed search results. Only studies published since 2000 were selected. The search was limited to publications written in English. Two independent reviewers (LA and IL) screened all retrieved records by title and abstract to identify potentially eligible studies. For articles meeting these criteria with full text available, the following further selection criteria were used: articles were excluded if they were off topic after investigating the full text. The entire flow and results of the literature research were finally checked by a third researcher (SN).

This systematic review was not registered in PROSPERO or any other public registry.

### 2.2. Data Extraction and Collection

After the selection procedure, the selected articles that met the inclusion criteria were analyzed by two reviewers (LA and IL) and finally verified by a third reviewer (SN). For each study, the following characteristics were collected: (first author name, year of publication, and method of study, namely in vivo and/or in vitro), name of the aptamer investigated, nucleic acid sequence, aptamer target, diagnostic application depending on whether the study used aptamers for diagnosis, and dissociation constant (Kd).

Given the predominantly preclinical/analytical nature of the included studies, a formal risk-of-bias tool was not considered directly applicable; instead, methodological limitations of individual studies (e.g., sample size, validation cohort, potential conflicts of interest) were critically discussed narratively.

### 2.3. Planning and Conducting the Review

The selected studies were classified based on the diagnostic modality employing the aptamer, distinguishing among imaging techniques, liquid biopsy applications, and aptamer-based sensing systems. This systematic review was conducted following the Preferred Reporting Items for Systematic Reviews and Meta-Analyses (PRISMA) statement (see Supplementary Materials Table S2).

## 3. Results

### 3.1. Study Selection

A total of 105 articles were retrieved from PubMed, Google Scholar, Web of Science, and Science Direct databases. After the removal of 30 duplicate articles (S1f), we performed a screening based on titles and abstracts of the remaining 75 articles; four records were review articles, the remaining eight only mentioned the words “aptamers” and/or “NSCLC” in the abstract, and another one did not focus on diagnostic application.

Screening by titles and abstracts yielded 62 potentially eligible articles, which were evaluated by their full text. Of these articles, 23 records were excluded because they did not investigate the role of aptamers in NSCLC diagnosis by having a therapeutic goal. Finally, 39 records were included for qualitative synthesis.

### 3.2. Aptamer-Based Imaging

Here, we will discuss various studies from 2000 to the present that address the use of aptamers for diagnostic purposes on tissues and cell lines. In this regard, 15 studies involve them in imaging and are summarized in Table 2.

In NSCLC, several studies have highlighted the role of aptamers for targeting clinically relevant biomarkers. For instance, in 2023 Thomas et al. explored the anti-EGFR 2′-FY RNA aptamer, MinE07, as a novel targeting tool for NSCLC. MinE07 retains its binding capacity to both wild-type and mutant EGFR and competes with clinically used monoclonal antibodies such as cetuximab and nimotuzumab. Moreover, to bind both EGFR and c-Met, the authors generated a bispecific aptamer linking MinE07 and CLN3, a 50-nt DNA aptamer that targets c-Met. This construct is functionalized with Atto647N and enhanced NSCLC cells labelling in vitro, although this advantage was not reproduced in vivo, likely due to heterogeneous c-Met expression and differences in tumor penetration. Overall, MinE07 emerges as a promising ligand for diagnostic and therapeutic applications in EGFR-mutant NSCLC, while further optimization is required to improve in vivo stability, targeting specificity, and translational potential [14]. Another study presented a reagent-less electrochemical capacitive biosensor for rapid and ultrasensitive EGFR detection in NSCLC. A specific EGFR aptamer, developed via SELEX, was immobilized on a chromium/gold interdigitated capacitor electrode (IDCE). The biosensor detects EGFR through capacitance changes upon binding, achieving label-free detection within 3 s and a limit of 0.005 ng/mL.

Validation was carried out using purified EGFR peptide solutions, human lung cancer cell lines (H1975, HCC827, A431) and K-Ras mutant transgenic mice, confirming strong affinity and selectivity of the aptamer both in vitro and in vivo. These findings highlight the biosensor’s potential as a portable, cost-effective tool for early NSCLC detection and future clinical diagnostics [15].

Along the same line, another work by Ren et al. introduces a novel DNA aptamer, 8–60, targeting PD-L1, a key biomarker in cancer diagnosis and immunotherapy, developed through the innovative REase-SELEX method, which combines restriction enzyme activity with the protein SELEX process to enhance sequence diversity and specificity; indeed, the restriction enzyme Alu I was not employed in its traditional role, but as a key component of the aptamer selection process. First, it acts as a selective filter: DNA sequences that do not bind the chosen target remain accessible to Alu I and are cleaved; thus, only the sequences that are able to bind the target protect their recognition site from enzymatic cleavage, thereby surviving and becoming enriched in the pool. Moreover, Alu I promoted extensive mutations within the DNA library. Thus, through this dual mechanism of target-dependent protection and mutation-driven diversification, the group generated novel DNA aptamer targeting PD-L1 showing nanomolar affinity for its target. Importantly, 8–60, functionalized with the fluorophore Cy5.5, was validated on clinical tissue samples, enabling reliable fluorescence imaging of PD-L1 expressions in NSCLC and malignant melanoma, as well as in normal tonsil tissue used as the diagnostic gold standard. These findings highlight 8–60 as a promising diagnostic probe for precise evaluation of PD-L1 expression levels, with potential applications in patient stratification for immunotherapy [16]. Recognizing the goodness of this target for diagnostic use in NSCLC, Li et al. performed a SELEX integrating three different types of this technique to generate a DNA aptamer specifically designed to recognize PD-L1. The novel selection strategy is called Modular-SELEX, and combines (1) MCP-SELEX, (2) Capture SELEX and (3) Tissue-SELEX. Through this multistep approach, the team generated a DNA aptamer named Clon-3, which exhibited nanomolar affinity and high specificity for PD-L1. Clon-3 was tested on tissue sections from normal human tonsils and NSCLC, the aptamer was able to visualize PD-L1 with fluorescence imaging using Cy5.5. The observed results were consistent with traditional immunohistochemistry, confirming that this approach could therefore represent an important step forward for diagnostic and imaging applications [17]. Cy5.5 was used also by Xu et al. in 2022 for a study presenting a strategy to build multivalent DNA–protein nanostructures for tumor imaging and targeted therapy. They used two different aptamers: AS1411, which targets nucleolin, a protein overexpressed on the membrane of various cancer cells, including lung carcinoma (LLC); and TD05, which binds to membrane immunoglobulin M (IgM) expressed on Ramos lymphoma cells. These aptamers act as the “recognition heads” of the nanostructure, ensuring that the nano systems selectively bind to cancer cells and were incorporated into programmable DNA nanowires through regions known as “sticky ends”. These sticky ends allow individual DNA strands to self-assemble into long, periodic nanowires. When mixed with protamine, the nanowires condensed into dense DNA nanospheres. For diagnostic imaging, the aptamer-functionalized nanospheres were labeled with the near-infrared fluorescent dye Cy5.5, using modified AS1411, which allows deep-tissue imaging in orthotopic and subcutaneous NSCLC murine models. In these animal models, Cy5.5-labeled nanospheres accumulated specifically at tumor sites and they also enabled visualization of metastatic lymph nodes, confirming the aptamers’ ability to guide the nanostructures to tumor tissues with remarkable precision in living organisms [18]. Taken together, these studies illustrate the rapid evolution of aptamer-based fluorescent probes for NSCLC diagnosis; building on these advances, recent works improve fluorescence efficiency and biocompatibility through the aptamer functionalization with quantum dots (QDs).

In this context, an innovative one-pot synthesis of Zn^2+^-doped CdTe QDs directly functionalized with DNA aptamers has been reported for tumor-targeted fluorescence diagnosis. The approach yields highly fluorescent, biocompatible nanoprobes capable of specific molecular recognition and efficient tumor imaging both in vitro and in vivo. DNA serves a dual role: as a stabilizing ligand through phosphorothioate linkages binding to the QDs, and as a functional aptamer domain targeting Mucin 1 (MUC1), a biomarker overexpressed in various cancers, including lung adenocarcinoma. The resulting nanoparticles are small (≈4 nm) and exhibit a high quantum yield (up to 80%), excellent photostability, and low cytotoxicity thanks to Zn-doping and DNA coating. Fluorescence assays confirmed that the DNA aptamers remain biologically active, capable of hybridizing with complementary sequences and specifically binding to MUC1-positive cells (A549), with negligible signal in negative controls. Confocal and in vivo imaging demonstrated strong tumor localization and minimal off-target fluorescence, confirming the potential of aptamer-functionalized QDs as precise fluorescent diagnostic tools. Thus, this work highlights the synergy between aptamer-based molecular recognition and quantum dot fluorescence [19]; however, it is worth noting that, despite the reduced toxicity conferred by Zn-doping, the CdTe core inherent to this and other Cd-based QD probes remains a translational concern: Cd^2+^ ions can leach upon nanocrystal degradation, triggering ROS generation, mitochondrial damage, and hepato/nephrotoxic effects in vivo [20], and complete elimination of this risk is not achievable while the emissive core remains Cd-based. Recent developments include activatable aptamer probes that emit fluorescence only upon target binding, a “recognition-after-labeling” method that enhances signal-to-noise ratios and minimizing background interference. This inversion of steps eliminates the common drawbacks of conventional systems, such as steric hindrance, QDs aggregation, and structural distortion of aptamers, which could compromise binding specificity. The S11e DNA aptamer, selected via SELEX for its high affinity toward NSCLC A549 cells, was used as the recognition element. The QDs were functionalized with biotin and subsequently coupled to streptavidin-modified S11e aptamer via high-affinity biotin–streptavidin interactions, forming a stable QD–aptamer complex suitable for fluorescence-based cell recognition. Confocal imaging and flow cytometry confirmed strong fluorescence localization on A549 cell membranes, while control cells or random DNA sequences exhibited negligible signals. Compared with conventional organic dyes (e.g., FITC), QDs provided a 2.25-fold higher signal-to-background ratio and superior photostability, with fluorescence decreasing by only 15% after 30 min of continuous illumination (versus 80% for FITC) [21]. Moreover, Wu et al. in 2018 showed that this aptamer-based fluorescence approach could become a valuable tool for early detection and clinical monitoring of NSCLC, generating an aptasensor combining the DNA aptamer S6, which specifically binds NSCLC A549 cells, with a supramolecule cyanine dye (cy-M) that produces a strong fluorescent signal upon binding to the aptamer’s G-quadruplex structure. The system showed excellent selectivity, distinguishing NSCLC cells from other lung cancer subtypes and non-cancerous cells. Importantly, it remained highly effective in complex biological samples, such as human serum and clinical pleural effusion, where it successfully identified NSCLC cells from patients [22]. Beyond fluorescence, several groups have developed standalone electrochemical and optical cytosensors for label-free NSCLC cell detection. Kivrak et al. immobilized an NSCLC-specific aptamer on polyacrylonitrile/polypyrrole nanofibers to build an electrochemical biosensing platform capable of detecting NSCLC cells with high sensitivity [23]. Similarly, Mir et al. developed an ultrasensitive nanobiosensor bioconjugate for cytosensing of human NSCLC cells [24], while Chen et al. more recently reported a simpler, reagent-based cytosensing platform in which the AS1411 aptamer prevents salt-induced gold nanoparticle aggregation, generating a colorimetric readout specific for A549 cells [25]. A complementary liquid crystal-based platform was also proposed by Khoshbin et al., who developed an aptasensing device for rapid monitoring of A549 cells, explicitly framed as a prototype for a future portable lung cancer diagnostic test kit [26]. Collectively, these platforms illustrate the breadth of transduction strategies—electrochemical, colorimetric, and liquid crystal-based—that have been explored for aptamer-based NSCLC cell recognition outside of fluorescence imaging. Aptamers can also be functionalized to allow PET imaging; Liu et al. reported the development and validation of a novel PET imaging probe, [^68^Ga] Ga-NOTA-SL1, designed to target the receptor tyrosine kinase c-Met, a biomarker often overexpressed in various tumors such as NSCLC; they used the SL1 DNA aptamer, which was chemically modified with a NOTA chelator and labeled with gallium-68 to allow PET imaging. In vitro confirmed selective binding to c-Met-expressing tumor cells. In vivo PET/CT imaging in mouse xenograft models demonstrated efficient tumor uptake and rapid clearance from the body, with tumor-to-muscle ratios around 2. Immunohistochemistry confirmed the correlation between c-Met expression and probe accumulation. [^68^Ga]Ga-NOTA-SL1 enables non-invasive visualization and quantification of c-Met expression, showing great potential as a precise diagnostic tool for cancer detection and a platform for future theranostic applications [27]. Moreover, aptamers can also detect unconventional tumoral markers such as epigenetic methylation and demethylation, which are crucial regulatory mechanisms influencing gene expression, genome stability, and tumor progression. Dysregulation of DNA and RNA demethylases, such as ALKBH3 and FTO, has been increasingly associated with various cancers, including NSCLC. However, traditional techniques used to quantify demethylase activity, such as HPLC, immunoassays, and isotope tracing, are often time-consuming, require complex labeling, or rely on costly antibodies. In this regard, Liu et al. present an elegant, label-free and dual-color biosensing strategy for the simultaneous detection of multiple DNA demethylases, exploiting the fluorescence activation of RNA aptamers Spinach and Mango, which can bind demethylases. These aptamers are small RNA sequences that emit fluorescence only upon binding their respective non-fluorescent fluorogens, DFHBI (green) and TO1-biotin (orange), thus functioning as molecular “light switches”. Upon binding DFHBI and TO1-biotin, respectively, these aptamers produce distinct green and orange fluorescence signals, allowing dual-color quantitative detection of ALKBH3 and FTO activities. Importantly, the authors validated the platform using clinical samples, successfully differentiating between healthy and NSCLC tissues based on fluorescence intensity [28]. Finally, a study also showed the possibilities to allow an aptamerbased penta-modal imaging (photoacoustic (PA), CT, optical coherence tomography (OCT), thermal (PTI), and upconversion luminescence (UCL)) offering comprehensive visualization of tumor structure, vasculature, metabolism, and temperature in real time. The study presented the development of a multifunctional nanoplatform named UCILA for precise diagnosis and therapy NSCLC. UCILA integrates upconversion nanoparticles (UCNPs) and the IR-1048 dye into an AS1411 aptamer-modified lipid nanostructure, enabling both tumor targeting and multimodal imaging in the second near-infrared (NIR-II) window. This nanoplatform provides accurate tumor monitoring, targeted ablation, and immune reinforcement in NSCLC treatment [29].

**Table 2:** Summary of studies employing aptamers for tissue-based imaging of NSCLC biomarkers.

Authors, Year’s Publication	Ref.	Type	Target	Biological Application	Analytical Platform
Thomas et al., 2023	[14]	DNA	EGFR, c-Met	Early NSCLC detection (in vivo)	Bispecific aptamer
Ganbold et al., 2025	[15]	DNA	EGFR	Early NSCLC detection (in vivo)	Aptamer immobilized on electrode
Ren et al., 2021	[16]	DNA	PD-L1	Early NSCLC detection (ex vivo)	Aptamer
Li et al., 2021	[17]	DNA	PD-L1	Early NSCLC detection (ex vivo)	Aptamer
Xu et al., 2022	[18]	DNA	Nucleolin, IgM	Early NSCLC and metastatic lymph node detection (in vivo)	Multivalent-protein nanostructure
Zhang et al., 2013	[19]	DNA	MUC-1	Early NSCLC detection (in vivo)	Zn^2+^-doped CdTe QDs
Wu et al., 2015	[21]	DNA	Target not identified (ligand of A549)	Early NSCLC detection (in vivo)	QD–aptamer complex
Wu et al., 2018	[22]	DNA	Target not identified (ligand of A549)	Early NSCLC detection (in vivo)	Aptamer + CyM
Kivrak et al., 2020	[23]	DNA	Target not identified (ligand of NSCLC)	Early NSCLC cell detection (in vitro)	Electrochemical biosensor (PAN/PPy nanofibers)
Mir et al., 2015	[24]	DNA	Target not identified	Early NSCLC cell detection (in vitro)	Nanobiosensor bioconjugate (cytosensing)
Chen et al., 2025	[25]	DNA	Nucleolin	Early NSCLC cell detection (in vitro)	AuNP colorimetric cytosensor
Khoshbin et al., 2024	[26]	DNA	Target not identified (ligand of A549)	Early NSCLC cell detection (in vitro)	Liquid crystal-decorated aptasensor
Liu et al., 2025	[27]	DNA	c-Met	Early NSCLC detection (in vivo)	Aptamer + gallium 68
Liu et al., 2024	[28]	DNA	ALKBH3, FTO	Early NSCLC detection (ex vivo)	Aptamer+ DFHBI and TO1
Xu et al., 2021	[29]	RNA	Nucleolin	Early NSCLC detection and tumor monitoring (in vivo)	Multimodal nanostructure (UCILA)

### 3.3. Aptamer-Based Liquid Biopsy

Since conventional tissue biopsies are invasive and often fail to capture tumor heterogeneity, the development of a sensitive liquid biopsy method to identify the tumor remains an important challenge. Here we summarize studies in which aptamers were used to detect biomarkers exclusively in biological fluids of NSCLC patients. The 24 selected (summarized in Table 3) articles can be divided into three major groups: aptamers detecting circulating tumor cells (nine articles), or exosomes (nine articles), or proteins (six articles).

#### 3.3.1. Circulating Tumor Cells

An earlier foundation for aptamer-based CTC capture in NSCLC was laid by Zamay et al., who developed DNA aptamers directly against native EpCAM using a competitive antibody-displacement selection strategy on primary lung tumor cells. The resulting aptamers showed nanomolar affinity for EpCAM-positive lung cancer cells, and their target specificity was confirmed by confocal microscopy and spectral profiling of lung cancer tissue. Importantly, these aptamers were subsequently applied to isolate and detect circulating tumor cells in peripheral blood samples from lung cancer patients, providing one of the earliest clinical demonstrations that aptamer-based EpCAM recognition can support CTC-based liquid biopsy in NSCLC [30].

Liu et al. developed a powerful electrochemical liquid biopsy platform for the detection of circulating tumor cells (CTCs) and their PD-L1 expression in patients with non-small cell lung cancer (NSCLC) simultaneously. The authors designed a dual-DNA aptamer nanoprobe composed of nitrogen-doped carbon quantum dots (N-CQDs) functionalized with the EpCAM (epithelial marker) aptamer SYL3C and the cell-surface vimentin (mesenchymal marker) aptamer ZY5C (dual-aptamer E/V-apt-N-CQDs), enabling efficient capture of heterogeneous circulating tumor cell (CTC) populations, including cells undergoing epithelial–mesenchymal transition (EMT). For PD-L1 detection, they developed a reagent-less electrochemical sensor based on the hairpin PD-L1 aptamer MJ5CP conjugated to gold nanoparticles (PD-L1-apt-AuNPs). Upon specific binding to PD-L1, the aptamer undergoes a conformational change that exposes a biotin moiety, generating a measurable electrochemical redox signal. The dual-aptamer E/V-apt-N-CQDs probe achieved capture efficiencies exceeding 90% for NSCLC-derived CTC models. The electrochemical platform showed high specificity and sensitivity, detecting recombinant human PD-L1 protein in PBS buffer over a concentration range of 2–10 ng/mL, with a limit of detection of 2 ng/mL. The assay was subsequently applied to quantify PD-L1 expression in lysates of captured CTCs and in blood samples from patients with non-small cell lung cancer (NSCLC) [31]. A similar DNA dual-aptamer strategy, simultaneously targeting EpCAM and vimentin, was used by Zuo et al. Here, the authors developed a wrinkled cellulose hydrogel interface that combines high biocompatibility and large surface area, providing an efficient bio interface for CTC capture. Captured CTCs are quantified using a glucometer-based readout, enabled by a glucose-loaded, gold–mesoporous silica nanoparticle probe (Au–G–MSN–Apt). The release of glucose upon recognition provides an enzymatically amplified signal measurable with a commercial glucometer, transforming CTC detection into a simple, rapid, and low-cost assay suitable for point-of-care applications [32].

This promising approach was further advanced by Xu et al., who developed a device that employs a biomimetic polycarbonate membrane as the capture interface, functionalized with EpCAM and vimentin for the simultaneous capture and phenotypic characterization of CTCs undergoing EMT. The device employs a biomimetic polycarbonate microfiltration membrane as the capture interface, enabling label-free enrichment of heterogeneous CTC populations based on size differences. Phenotypic discrimination is achieved using two distinct CdTe quantum dots (QDs) conjugated with aptamers targeting EpCAM and vimentin, respectively, allowing the simultaneous visualization and quantification of epithelial and mesenchymal CTC subpopulations. Furthermore, the system was successfully validated using whole-blood samples from 94 patients with NSCLC, revealing significant associations between mesenchymal CTCs, disease stage, and metastatic status [33].

The use of aptamers for highly sensitive CTC detection was further explored by Liv et al., who developed a surface-enhanced Raman spectroscopy (SERS)-based platform for the detection of single circulating tumor cells in blood samples. The system combines a microporous parylene membrane for size-based cell enrichment with an aptamer-functionalized SERS nanoprobe for specific tumor cell recognition. The nanoprobe consists of gold nanoparticles modified with the Raman reporter 4-mercaptobenzoic acid (4-MBA) and an A549 cell-specific DNA aptamer, enabling selective identification of NSCLC cells. Following membrane filtration, captured cells were analyzed by single-cell Raman mapping, and tumor cells were distinguished from residual white blood cells based on their characteristic Raman signal intensity. The platform demonstrated high sensitivity and specificity, successfully detecting as few as 20 tumor cells in blood samples, with recovery and recognition rates exceeding 90%. Moreover, the aptamer-SERS probe efficiently discriminated against A549 cells from both white blood cells and non-target cancer cells, highlighting its potential as a rapid and cost-effective approach for liquid biopsy applications and early cancer detection [34].

Another work describes the efficient capture and genetic analysis of CTCs from NSCLC patients, a microwell-assisted multi-aptamer immunomagnetic platform (MMAIP). The system integrates magnetic nanoparticles functionalized with multiple aptamers (apt-MNPs) that selectively recognize distinct subpopulations of lung cancer cells, such as cisplatin-sensitive (A549) and cisplatin-resistant (A549-D) cell lines. The combination of two aptamers with different binding affinities allows synergistic co-recognition and maintains high capture efficiency across heterogeneous tumor cell types. Following magnetic capture, the cells are purified through a microwell chip. This markedly improves capture purity by spatially isolating rare CTCs from abundant white blood cells and enables downstream single-cell genetic analysis. Indeed, the authors, by using this approach, successfully detected clinically relevant mutations in KRAS and EGFR from CTCs isolated both from model systems and NSCLC patient blood samples [35]. Zhao et al. conducted a translational study aimed at improving the capture of CTCs in NSCLC using a microfluidic device functionalized with rationally designed aptamer cocktails. Even if the authors conducted extensive in vitro optimization using multiple NSCLC cell lines, the clinical validation remains exploratory. The authors first identified four aptamers suitable for synergistic combination (Ap1–Ap4). Using silicon nanowire substrate (SiNS) microfluidic chips, they demonstrated that dual- and tri-aptamer cocktails enhanced capture efficiency compared with single aptamers, and that different cocktails exhibited differential capture patterns depending on tumor cell subtype [36]. This platform was a useful and robust proof-of-concept for Qingfeng Lin. that very recently proposed a cocktail of different aptamers. In this case, the microfluidic platform integrates a three-zone PDMS microchip for cell sorting with a PEC sensing interface based on CuInS_2_/AuNP nanocomposites, functionalized with two specific aptamers: RA16 for NCI–H460 cells and EGFR aptamer for NCI–H1650 cells. This dual-aptamer configuration enables targeted capture of distinct NSCLC cell subtypes directly from blood samples, reflecting tumor heterogeneity. Under optimized conditions, the system achieved separation efficiency and purity of 83.4% and 76.8%, respectively, with detection limits of 15–16 cells/mL [37]. Zhu et al. introduced a highly sensitive, amplification-based mass spectrometric assay for quantifying PD-L1 on platelets, establishing its clinical potential as a liquid biomarker for immunotherapy in NSCLC. They designed a photo-cleavable mass-tagged self-assembled (SAMT) nanoprobe, composed of a PD-L1-specific aptamer, mass tags, and an amphiphilic polymer. Upon binding to PD-L1 on platelets, UV exposure releases mass tags, which can then be quantified by mass spectrometry. The authors quantified PD-L1 expression in both cancer cells and platelets from 46 NSCLC patients and 19 healthy donors, achieving a detection limit of 10 femtomolar. PD-L1 levels on platelets were markedly higher in NSCLC patients, particularly in those with advanced tumor stages and lymph node invasion [38].

#### 3.3.2. Exosomes

Here, we fully investigate aptamers targeting exosomes in NSCLC. Among the five selected articles, four of them develop aptasensor for an easy and sensitive detection of exosomes.

Very recently, Chang et al. described the development of an ultrasensitive nanomaterial-enhanced electrochemical aptasensor for detecting PD-L1-positive exosomes. The in vitro results show a very low detection limit (36 particles/mL), and good stability. The clinical validation is minimal, comprising only 10 serum samples from NSCLC patients and healthy volunteers [39]. In the same way, Wang et al. developed a microliter-scale organic electrochemical transistor (OECT) biosensor functionalized with a PD-L1-specific DNA aptamer immobilized on a high-surface-area carbon nanofiber–gold nanoparticle (CNFs–GNPs) gate. The device was engineered to detect PD-L1-positive exosomes with high sensitivity. Analytical evaluation using A549-derived exosomes demonstrated a 10 pg/mL detection limit, rapid response, and good stability. Clinical feasibility was assessed using 21 plasma samples (18 PD-L1-positive NSCLC patients, three healthy controls), where the sensor clearly differentiated patient samples based on current-response shifts. The study provides proof-of-concept evidence supporting aptamer-based OECT platforms for PD-L1 exosome detection, although conclusions are limited by the small, highly selected cohort and absence of disease controls [40]. A complementary signal-amplification strategy for exosomal PD-L1 detection was reported by Li et al., who developed a single-step, aptamer-activated cascade primer exchange reaction (PER) system generating branched DNA nanostructures for highly sensitive imaging of exosomal PD-L1. Upon aptamer binding to PD-L1 on the exosome surface, a cascade of autonomous primer exchange reactions is triggered, producing a dendritic DNA nanostructure that carries multiple fluorophore-labeled reporter strands per binding event, thereby amplifying the fluorescence signal without enzymatic amplification or thermal cycling. This one-step, isothermal approach markedly reduces assay complexity compared with conventional multistep exosome detection protocols, while achieving high sensitivity for PD-L1-positive exosomes [41]. Wang et al. developed an electrochemical aptasensor for exosome detection based on a methylene blue (MB)-stained single-stranded DNA aptamer specific for Ep-CAM, SYL3C. In the absence of EpCAM-positive exosomes, MB binds to guanine residues of the aptamer, producing a strong electrochemical signal. When EpCAM-positive exosomes are present, aptamer binding induces a conformational change that reduces MB interaction and electron transfer, leading to a signal decrease. This “electronic switch” enables sensitive, label-free detection of exosomes directly from serum samples, achieving ultra-low detection limits [42].

Xu et al. developed a dual-mode aptasensor based on gold nanoparticles (AuNPs) and a more complex hybrid structure, called Co@g-C3N4@PB; both are functionalized with modified aptamers targeting the CD63 protein, a common exosomal marker. The AuNPs act as gold electrodes that generate an electrochemical readout by binding exosomes through CD63, while the second nanocarrier consists of g-C3N4 nanosheets doped with cobalt single-atom nanozymes (Co-SANs) to improve catalytic activity and electrical conductivity, forming Co@g-C3N4; Prussian Blue nanoparticles (PB) are then incorporated to enhance the electrochemical and fluorescent signal output. Using this platform, the authors analyzed serum-derived exosomes from 42 subjects (21 NSCLC patients and 21 healthy controls, all over 60), detecting stronger signals in cancer samples compared to healthy controls, reflecting the higher abundance of exosomes in NSCLC patients [43].

A related dual-mode strategy for NSCLC exosome detection was reported by Xu et al., who engineered artificial nanoenzymes based on oxidized iridium nanoparticles self-coupled with horseradish peroxidase (IrO2NPs@HRP), functionalized with the CD63 aptamer. This platform enabled both a naked-eye colorimetric readout and a quantitative electrochemical signal, and was additionally capable of detecting EGFR protein on the surface of captured NSCLC exosomes, adding a layer of molecular characterization beyond simple exosome counting [44]. A distinct logic-gate-based approach was proposed by Meng et al., who developed a ratiometric electrochemical “OR gate” assay capable of generating a positive signal in response to either of two independent NSCLC exosomal markers, thereby improving detection robustness against marker heterogeneity across exosome subpopulations [45].

The authors introduce an integrated concentration and determination system of exosomes (ICDSE) that combines exosome isolation and miRNA detection in a single, streamlined assay, addressing the limitations of traditional ultracentrifugation and costly microfluidic approaches. The ICDSE system employs aptamer-engineered erythrocytes (red blood cells, RBCs) to capture exosomes directly from plasma using an aptamer targeting CD63. Exosomes bound to engineered RBCs can be easily separated by low-speed centrifugation and released through DNase treatment; for molecular detection, the authors coupled this enrichment step with a plasmonic biosensor based on a self-assembled quadruplet supramolecular DNA dendrimer integrated with a zirconium-based metal–organic framework (Zr-MOF). This structure provides significant signal amplification, enabling ultrasensitive detection of exosomal miR-155—a known NSCLC biomarker—with a limit of detection as low as 2.03 femtomolar [46].

The study of Yu et al. reports the development of a LFAA (Lateral Flow Aptamer Assay) strip for rapid and low-cost detection of NSCLC A549-derived exosomes. They generated a competitive LFAA format where the appearance of the red line on the test strip is inversely related to the presence of exosomes. The test line (negative control) is coated with a complementary strand of the CD63 aptamer immobilized via streptavidin-biotin. Gold nanoparticles functionalized with CD63 aptamers flow along the strip with the sample. When exosomes are present in the sample, they bind to the aptamers on the gold nanoparticles, preventing the nanoparticles from binding to the T line. As a result, the T line appears lighter or even disappears entirely. Conversely, when exosomes are absent, there is no competition for the aptamer-functionalized nanoparticles. These free nanoparticles readily bind to the complementary strand on the T line, generating a visible red band. The strip showed specificity for A549 exosomes, effectively distinguishing them from non-specific exosomes and other molecules [47].

#### 3.3.3. Protein

This study describes the development and validation of a multi-protein plasma biomarker panel for detecting NSCLC using a high-throughput aptamer-based proteomic platform. The study offers an initial proof-of-concept for a 7-protein aptamer-based classifier for NSCLC, but the methodological constraints substantially limit confidence in the reported diagnostic accuracy [48]. In a second study, the same authors provide preliminary evidence supporting the diagnostic potential of the AptoDetect™-Lung 7-protein panel in distinguishing NSCLC from benign nodules. Here, the sample size is large (*n* = 400) and the inclusion of CT-characterized benign nodules reflects a clinically relevant control group; the design inherently carries a high risk of spectrum and selection bias. Controls were significantly younger and had substantially smaller nodules compared with cases, introducing baseline imbalances that may artificially inflate the test’s specificity. Performance for early-stage disease remained limited (AUC 0.70), and no comparison with validated clinical risk models (e.g., Brock, Lung-RADS) was conducted. Follow-up of benign nodules for only one year may not exclude slow-growing adenocarcinomas. Additionally, the presence of industry affiliation among some authors raises the possibility of conflict of interest. Overall, while the panel demonstrated promising specificity in high-risk individuals, the lack of prospective validation in a true LDCT screening cohort limits the generalizability of these findings [49]. The study by Ostroff et al. [50] represents a large-scale clinical application of an aptamer-based proteomic technology for the discovery of blood biomarkers for the early detection of NSCLC. Using serum samples from 1326 subjects across four independent cohorts, the authors quantified 813 proteins and identified 44 candidate biomarkers. From these, a 12-protein panel, including cadherin-1, CD30 ligand, endostatin, HSP90α, LRIG3, MIP-4, pleiotrophin, PRKCI, RGM-C, SCF-sR, sL-selectin, and YES, was developed, achieving 91% sensitivity and 84% specificity in training and 89% sensitivity and 83% specificity in an independent validation set. They developed this platform following the methods of Gold et al. [51]; briefly, the aptamer-based multiplex proteomic assay converts a complex biological sample into a quantitative protein profile using modified DNA aptamers with high specificity. Aptamers bind their target proteins in solution, forming stable complexes that are sequentially captured, purified, and released through biotin–streptavidin interactions and a kinetic challenge with dextran sulfate to remove non-specific bindings. Each aptamer is then hybridized to its complementary sequence on a DNA microarray, where fluorescence intensity reflects the abundance of the corresponding protein, enabling simultaneous and highly sensitive quantification of hundreds of proteins [50]. A related but distinct proteomic strategy was proposed by Wang et al., who developed a machine learning-boosted serum protein fishing platform for the diagnostic classification of small pulmonary nodules. Using magnetic nanoparticles to capture a protein corona from patient serum, the platform combines aptamer-based protein enrichment with a machine learning classifier trained to distinguish malignant from benign nodules, addressing a clinically pressing gap in the triage of indeterminate nodules detected by low-dose CT screening [52]. In the same way, Sun and colleagues developed a novel surface-enhanced Raman scattering (SERS)-based biosensor for the ultrasensitive detection of cytochrome c (Cyt c) in the serum of patients with NSCLC. Cyt c, a key regulator of early apoptosis, has been identified as a promising biomarker for NSCLC, as its serum levels are significantly reduced in patients at diagnosis, reflecting impaired apoptotic pathways and enhanced tumor progression. Traditional diagnostic approaches are often limited by low sensitivity, high cost, and the difficulty of detecting low-abundance biomarkers, motivating the development of more sensitive and practical detection platforms. In this study, the authors designed a biosensor using gold nanourchins (GNUs) assembled on hydrophobic filter paper. The GNUs were functionalized with Cyt c-specific aptamers and complementary DNA labeled with a Cy5 Raman reporter. Upon binding of Cyt c to the aptamer, the complementary strand detached, leading to a measurable decrease in the SERS signal. Finite difference time domain (FDTD) simulations confirmed that the sharp protrusions and aggregation of the GNUs generated dense “hot spots,” producing strong electromagnetic field enhancements essential for SERS sensitivity. The hydrophobic modification of the paper, achieved via alkyl ketene dimer treatment, improved nanoparticle deposition and analyte retention, enhancing signal uniformity and reproducibility. The biosensor demonstrated excellent sensitivity, with detection limits as low as 1.148 pg/mL in buffer and 1.79 pg/mL in human serum, and its performance was consistent with conventional ELISA measurements [53].

The study “Proteome Fishing for CRISPR/Cas12a-Based Orthogonal Multiplex Aptamer Sensing” introduces an innovative diagnostic approach, termed COMPASS, that integrates nanoparticle-based proteome fishing with CRISPR/Cas12a technology for multiplex serum biomarker detection. The term “orthogonal multiplex” refers to the fact that multiple CRISPR/Cas12a systems, each guided by a distinct crRNA, can operate simultaneously without cross-interference. This enables the detection of several proteins within the same sample at once. The authors developed a system that uses magnetic nanoparticles (MNPs) to capture a protein corona (PC) from patient serum. This protein layer serves as a molecular “fishing net” that enriches relevant biomarkers. A set of aptamers, specific for clinically relevant proteins such as HE4, NSE, AFP, and VEGF165, was then applied to recognize these corona-associated biomarkers. When the aptamer interacts with its target protein, this binding event triggers the activation of a DNA fragment, used as the recognition target for a CRISPR/Cas12a system. Once Cas12a recognizes the trigger DNA, it becomes activated and exhibits its collateral cleavage activity, cutting a fluorescent reporter probe and thereby generating a detectable signal. This method achieved remarkable diagnostic accuracy for NSCLC [54].

**Table 3:** Summary of studies employing aptamers for liquid biopsy of NSCLC biomarkers.

Authors, Year’s Publication	Ref.	Type	Target	Biological Application	Analytical Platform
Zamay et al., 2019	[30]	DNA	EpCAM	Isolation of CTCs from patient blood	Antibody-displacement selected aptamers
Liu Y et al., 2025	[31]	DNA	EpCAM, vimentin, PD-L1	Capture of epithelial + mesenchymal CTCs; PD-L1 quantification on CTCs	Dual-aptamer N-CQDs + electrochemical PD-L1-apt-AuNPs sensor
Zuo et al., 2023	[32]	DNA	EpCAM, vimentin	Capture of heterogeneous CTCs (point-of-care)	Wrinkled cellulose hydrogel + Au-G-MSN-Apt glucometer readout
Xu H et al., 2024	[33]	DNA	EpCAM, vimentin	CTC phenotype detection + EMT tracking	Biomimetic polycarbonate membrane (PDMS chip) + CdTe QDs
Lv et al., 2024	[34]	DNA	Target not identified (ligand of A549)	Single-CTC detection	Au-4-MBA SERS nanoprobe + microporous parylene membrane
Dong et al., 2018	[35]	DNA	Multiple A549-specific aptamers	CTC capture + single-cell genetic profiling (KRAS, EGFR)	Aptamer-MNPs + microwell chip (MMAIP)
Zhao et al., 2016	[36]	DNA	Target not identified (ligand of NSCLC)	Enhanced/differential CTC capture	SiNS microfluidic chip + aptamer cocktail
Lin Q et al., 2026	[37]	DNA	EGFR	Subtype-specific CTC detection	Three-zone PDMS microchip + PEC sensor and fluorescence
Zhu et al., 2024	[38]	DNA	PD-L1	Detection of PD-L1 on tumor-educated platelets	SAMT nanoprobe + LC-MS
Chang et al., 2023	[39]	DNA	PD-L1	Detection of PD-L1-positive exosomes	Electrochemical aptasensor (PdCuB nanotag)
Wang Y et al., 2025	[40]	DNA	PD-L1	Detection of PD-L1-positive exosomes from micro-liters of blood	OECT (CNFs-GNPs)
Li X et al., 2022	[41]	DNA	PD-L1	Imaging of exosomal PD-L1	Aptamer-activated cascade primer exchange reaction (PER)
Wang Y et al., 2023	[42]	DNA	EpCAM	Exosome detection (reagent-less electronic switch)	Electrode + methylene blue-stained SYL3C aptamer
Xu X et al., 2024 (ACS Omega)	[43]	DNA	CD63	NSCLC exosome capture + dual detection	Electrochemical + fluorescence (Co@g-C3N4@PB)
Xu X et al., 2024 (Anal Sci)	[44]	DNA	CD63	NSCLC exosome capture + dual detection	Dual-mode colorimetric/electrochemical (IrO2NPs@HRP)
Meng F et al., 2023	[45]	DNA	EpCam, CEA	Exosome detection via OR-gate logic	Ratiometric electrochemical assay
Fan et al., 2023	[46]	DNA	CD63	High-throughput exosome capture + exosomal miRNA detection (ICDSE)	Aptamer-engineered erythrocytes + Zr-MOF biosensor
Yu et al., 2020	[47]	DNA	CD63	Detection of A549-derived exosomes (point-of-care)	LFAA strip (AuNP + CD63 aptamer)
Jung et al., 2017	[48]	SOMAmer	Plasma proteins (~1000 screened)	Proteomic profiling; 7-protein diagnostic panel	Aptamer-based proteomics (SOMAscan)
Jung et al., 2018	[49]	SOMAmer	EGFR1, MMP7, CA6, KIT, CRP, C9, SERPINA3	Validation of the AptoDetect™-Lung panel	Aptamer-based proteomics (AptoDetect-Lung)
Ostroff et al., 2010	[50]	SOMAmer	Cadherin-1, CD30 ligand, Endostatina, HSP90α, LRIG3, MIP-4, Pleiotrofina, PRKCI, RGM-C, SCF-sR, sL-selectina, YES	Serum biomarker discovery for early NSCLC detection	Aptamer-based multiplex proteomics
Wang M et al., 2024	[52]	DNA	Serum protein corona (ML classifier)	Diagnostic classification of small pulmonary nodules	Magnetic nanoparticle protein-fishing + ML classifier
Sun et al., 2020	[53]	DNA	Cytochrome c	Ultrasensitive detection of Cyt c in NSCLC serum	SERS biosensor (gold nanourchins)
Li S et al., 2024	[54]	DNA	HE4, NSE, AFP, VEGF165	Multiplex serum biomarker detection (COMPASS)	Magnetic nanoparticle protein-fishing + CRISPR/Cas12a

### 3.4. Discussion

Non-small cell lung cancer (NSCLC) remains one of the most prevalent and deadliest forms of cancer globally, making early diagnosis, prognosis prediction, and targeted therapies critical for improving patient outcomes. While the field has seen considerable advancements in molecular diagnostics, the use of aptamers in NSCLC diagnosis is still in its early stages, despite promising preclinical results, primarily due to challenges in target identification and the dominance of antibodies in the pharmaceutical market. Aptamers, due to their ability to bind targets with high specificity and affinity, offer a significant advantage over traditional antibodies in terms of stability, easy modification, and non-toxicity. The findings of this review underscore the growing potential of aptamers as diagnostic tools for NSCLC; indeed, many studies have explored their use in both imaging techniques and liquid biopsy applications.

However, considered individually, the studies discussed above generally demonstrate promising analytical performance, but a broader evaluation of the field reveals important limitations that currently hinder clinical translation. Different aptamer-based diagnostic platforms have shown encouraging results in terms of target detection, sensitivity, and specificity, particularly in preclinical models and very limited clinical cohorts. Nevertheless, most approaches still require further validation in larger, independent, and prospective patient populations.

The development of aptamer-based technologies has progressed rapidly, but clinical validation remains a critical challenge for their successful integration into NSCLC diagnostic workflows.

This may also be attributable to the clinical implementation of aptamer-based diagnostic platforms in the healthcare systems and diagnostic industry, which are largely built around antibody-based technologies. Antibody-based assays are already well established in clinical laboratories, supported by standardized protocols, validated workflows, dedicated instrumentation, and regulatory approval [55]. Consequently, despite the numerous advantages offered by aptamers, including chemical synthesis, high reproducibility, and ease of modification, the transition from antibody- to aptamer-based diagnostics may require substantial investments in infrastructure, validation, and clinical implementation. Therefore, the widespread adoption of aptamer technologies will likely depend not only on their analytical and clinical performance, but also on demonstrating clear economic and practical advantages over existing diagnostic approaches.

Moreover, an additional factor that may contribute to the limited clinical translation of aptamer-based diagnostics is the choice of molecular targets themselves. Although many biomarkers are suitable for target recognition and analytical detection, not all of them provide equivalent clinical information when evaluated as standalone diagnostic markers. Some targets may represent actionable molecular alterations or biologically informative features of the tumor, whereas others require integration with additional molecular, pathological, or clinical parameters to acquire meaningful diagnostic value. Consequently, the success of aptamer-based platforms relies not only on the development of highly sensitive recognition technologies, but also on the rational selection and contextual interpretation of the targeted biomarkers.

For example, EGFR represents a biologically and clinically relevant target, as its alterations can drive tumor progression and influence therapeutic decisions [4]. Conversely, PD-L1 expression reflects a dynamic interaction between tumor cells and the immune microenvironment, being influenced by both intrinsic oncogenic pathways and adaptive immune responses; therefore, its levels may vary among NSCLC subtypes, tumor regions, and treatment stages [5]. Similarly, c-Met activation has been associated with tumor aggressiveness and resistance mechanisms, particularly in the context of targeted therapies, highlighting its potential value beyond simple tumor detection [7]. Other biomarkers, such as EpCAM and MUC1, mainly reflect epithelial tumor features, whereas markers such as CD63 are broadly associated with extracellular vesicles and should therefore be interpreted with caution regarding disease specificity [56]. Likewise, the accessibility of nucleolin on the cancer cell surface, which enables its recognition by specific aptamers, is linked to its altered cellular localization during tumorigenesis [57].

Therefore, distinguishing between biomarkers that provide mechanistic and clinically actionable information and those primarily used as capture elements is essential for evaluating the real translational potential of aptamer-based diagnostic platforms [58].

In parallel with target selection, the material composition of the diagnostic probe itself represents an equally important, yet often overlooked, determinant of clinical translatability. Beyond conjugation chemistry, the choice of nanocarrier also carries direct implications for biocompatibility and clinical translatability, and these should not be overlooked when designing aptamer-based diagnostic platforms. Gold nanoparticles benefit from decades of biomedical use and are generally regarded as chemically inert and well tolerated, representing the most clinically mature metallic scaffold [59].

Cadmium-based quantum dots (e.g., CdTe, CdSe), by contrast, raise more substantial safety concerns: cadmium is a recognized human carcinogen, and Cd^2+^ ions can be released upon nanocrystal degradation, triggering oxidative stress, mitochondrial dysfunction, and hepato/nephrotoxic effects in vivo; Zn-doping or ZnS shell coating mitigates but does not eliminate this risk, since the emissive core remains Cd-based [20]. Cobalt-containing nanomaterials, used for their catalytic and magnetic properties, have shown variable cytotoxicity profiles depending on particle size, coating, and dose, with oxidative damage reported in several cell models, indicating that their long-term safety cannot be assumed a priori [60]. Prussian Blue nanoparticles are comparatively favored from a translational standpoint, as this compound is already clinically approved as an antidote for radioactive cesium and thallium poisoning, supporting a more established safety profile [61]. Zirconium-based metal–organic frameworks (Zr-MOFs), although promising for signal amplification due to their high surface area and tunable porosity, remain a comparatively newer class of nanomaterial whose long-term biodegradation, clearance kinetics, and chronic toxicity in vivo are still incompletely characterized [62]. Taken together, these considerations indicate that the nanomaterial component of an aptamer-based probe is not a neutral analytical accessory, but a variable that materially affects the translational feasibility of the resulting diagnostic platform.

Looking forward, several concrete steps could accelerate the translation of aptamer-based diagnostics for NSCLC. First, some clinically relevant biomarkers used in current guidelines, such as ALK and ROS1 rearrangements, remain essentially “orphan” targets for which no validated diagnostic aptamer has yet been reported, representing a clear opportunity for future SELEX campaigns [4]. Moreover, more encouragingly, aptamers are not a purely preclinical curiosity: pegaptanib, an anti-VEGF aptamer, was approved by the FDA in 2004 for age-related macular degeneration and remains, together with the more recently approved avacincaptad pegol, one of only two aptamer drugs in clinical use [63,64], while AS1411, the nucleolin-targeting aptamer discussed throughout this review for NSCLC tissue imaging, has already completed phase II clinical trials in metastatic renal cell carcinoma and relapsed acute myeloid leukemia [65,66]. This existing clinical experience with AS1411 in other tumor types is a meaningful precedent that could directly inform and accelerate its translational development for NSCLC specifically, rather than requiring translation to start from scratch.

Taken together, the evidence discussed in this review highlights both the significant progress and the remaining challenges in the development of aptamer-based diagnostic platforms for NSCLC. Although the field has generated a wide range of innovative approaches for biomarker detection, further efforts are needed to bridge the gap between analytical performance and clinical applicability. Future studies should focus on the validation of biologically relevant targets, the integration of multiple biomarkers to better reflect NSCLC heterogeneity, and the evaluation of translational aspects of nanomaterial-based platforms.

## 4. Conclusions

In conclusion, while the clinical integration of aptamers in NSCLC diagnosis remains a work in progress, the field holds substantial promise. Realizing this potential will depend not only on continued technical refinement, but on the rational selection of clinically actionable targets, careful attention to the biocompatibility of the nanomaterials employed, and strategies to overcome the practical and economic barriers posed by an antibody-dominated diagnostic industry.

Taken together, the growing availability of targeted therapies and the ongoing refinement of aptamer-based technologies are likely to establish aptamers as valuable diagnostic tools within the evolving NSCLC molecular testing landscape.

## Figures and Tables

**Table 1:** Comparison of key biochemical and translational features between aptamers and antibodies.

Feature	Aptamers	Antibodies
Production	Chemical synthesis (SELEX-derived sequence)	Cell culture/animal immunization
Size	~8–25 kDa	~150 kDa (IgG)
Thermal/chemical stability	High; tolerates repeated denaturation-renaturation	Limited; irreversible denaturation
Chemical modification	Site-specific, straightforward	More complex, less site-specific
Batch variability	Very low	Low
Cost	Lower	Higher
Development time	Weeks	Months
Stability	High; tolerates temperature and pH changes	Lower; typically requires cold-chain storage
Chemical modification	Simple and site-specific	More complex
Immunogenicity	Negligible	Possible
Target range	Proteins, cells, exosomes, small molecules	Mainly proteins
Tissue penetration/Clearance	Rapid, favored by small size)	Slower, larger size limits penetration
Clinical validation	Emerging	Extensive
Regulatory approval	Limited	Well established
Main limitation	Susceptibility to nuclease degradation, unless chemically modified	Higher production cost and more complex protein engineering

## Data Availability

The original contributions presented in this study are included in the article/Supplementary Materials. Further inquiries can be directed to the corresponding authors.

## Supplementary Materials

The following supporting information can be downloaded at: https://www.mdpi.com/article/10.3390/ijms27156957/s1.

## Abbreviations

The following abbreviations are used in this manuscript:

4-MBA	4-Mercaptobenzoic Acid
AFP	Alpha-Fetoprotein
ALK	Anaplastic Lymphoma Kinase
Alu I	Arthrobacter luteus Restriction Enzyme I
AS1411	Anti-Nucleolin DNA Aptamer
AUC	Area Under the Curve
AuNPs	Gold Nanoparticles
BRAF	B-Raf Proto-Oncogene
CAR-NK	Chimeric Antigen Receptor Natural Killer
CD63	Cluster of Differentiation 63
c-Met	Mesenchymal–Epithelial Transition Factor
CNFs-GNPs	Carbon Nanofibers-Gold Nanoparticles
COMPASS	CRISPR/Cas12a-Based Orthogonal Multiplex Aptamer Sensing System
CRISPR	Clustered Regularly Interspaced Short Palindromic Repeats
CT	Computed Tomography
CTCs	Circulating Tumor Cells
Cyt c	Cytochrome c
Cy5.5	Cyanine 5.5 Fluorophore
DFHBI	3,5-Difluoro-4-hydroxybenzylidene Imidazolinone
DNA	Deoxyribonucleic Acid
EDC	1-Ethyl-3-(3-Dimethylaminopropyl)Carbodiimide
EGFR	Epidermal Growth Factor Receptor
ELISA	Enzyme-Linked Immunosorbent Assay
EMT	Epithelial–Mesenchymal Transition
EpCAM	Epithelial Cell Adhesion Molecule
FDTD	Finite-Difference Time Domain
FITC	Fluorescein Isothiocyanate
FTO	Fat Mass and Obesity-Associated Protein
GNUs	Gold Nanourchins
HE4	Human Epididymis Protein 4
HPLC	High-Performance Liquid Chromatography
HRP	Horseradish Peroxidase
ICDSE	Integrated Concentration and Determination System of Exosomes
IDCE	Interdigitated Capacitor Electrode
IgM	Immunoglobulin M
IrO2NPs	Iridium Oxide Nanoparticles
Kd	Dissociation Constant
KRAS	Kirsten Rat Sarcoma Viral Oncogene Homolog
LC	Lung Cancer
LC-MS	Liquid Chromatography–Mass Spectrometry
LDCT	Low-Dose Computed Tomography
LFAA	Lateral Flow Aptamer Assay
MB	Methylene Blue
MCP-SELEX	Multi-Cellular Protein SELEX
MET	MET Proto-Oncogene, Receptor Tyrosine Kinase
miRNA	MicroRNA
MNPs	Magnetic Nanoparticles
MSN	Mesoporous Silica Nanoparticle
MUC1	Mucin 1
N-CQDs	Nitrogen-Doped Carbon Quantum Dots
NIR-II	Second Near-Infrared Window
NHS	N-Hydroxysuccinimide
NOTA	1,4,7-Triazacyclononane-1,4,7-Triacetic Acid
NSCLC	Non-Small Cell Lung Cancer
NSE	Neuron-Specific Enolase
NTRK	Neurotrophic Tyrosine Receptor Kinase
OECT	Organic Electrochemical Transistor
OCT	Optical Coherence Tomography
PA	Photoacoustic Imaging
PBS	Phosphate-Buffered Saline
PC	Protein Corona
PD-1	Programmed Cell Death Protein 1
PD-L1	Programmed Death-Ligand 1
PDMS	Polydimethylsiloxane
PEC	Photoelectrochemical
PER	Primer Exchange Reaction
PET	Positron Emission Tomography
PMCID	PubMed Central Identifier
PMID	PubMed Identifier
PRISMA	Preferred Reporting Items for Systematic Reviews and Meta-Analyses
PTI	Photothermal Imaging
QDs	Quantum Dots
RBCs	Red Blood Cells
REase-SELEX	Restriction Enzyme-Assisted SELEX
RET	Rearranged During Transfection
RNA	Ribonucleic Acid
ROS1	ROS Proto-Oncogene 1
SAMT	Self-Assembled Mass-Tagged
SCF	Stem Cell Factor
SELEX	Systematic Evolution of Ligands by Exponential Enrichment
SERS	Surface-Enhanced Raman Spectroscopy
SiNS	Silicon Nanowire Substrate
SPGE	Screen-Printed Gold Electrode
TTF-1	Thyroid Transcription Factor-1
UCL	Upconversion Luminescence
UCILA	Upconversion Nanoparticle/IR-1048 Lipid Aptamer Nanoplatform
UCNPs	Upconversion Nanoparticles
UV	Ultraviolet
VEGF	Vascular Endothelial Growth Factor
Zr-MOF	Zirconium-Based Metal–Organic Framework
