# Supplementary material for: Advances in Aptamer Diagnostics Targeting Non-Small-Cell Lung Cancer: A Systematic Review
Source: Int J Mol Sci. 2026 Aug 3;27(15):6957. doi: 10.3390/ijms27156957 (PMC13467519; doi:10.3390/ijms27156957)
Supplement: Supplementary file 1 [file ijms-27-06957-s001.zip › S1f.pdf]

PMID	Study	Position
39929085	Liu Y et al., 2025	diagnosis #1, liquid_biopsy #1, sensor #1, capture #1
36270080	Fan Y et al., 2023	diagnosis #6, liquid_biopsy #2, sensor #2
38698606	Xu H et al., 2024	diagnosis #36, liquid_biopsy #6, capture #4
36562728	Zuo Y et al., 2023	diagnosis #44, liquid_biopsy #5, capture #6
40706481	Lin Q et al., 2026	diagnosis #50, imaging #18, capture #7
39437325	Ping W et al., 2024	diagnosis #3, imaging #2
40209314	Kejamurthy P et al., 2025	diagnosis #12, imaging #5
32935151	Kivrak E et al., 2020	diagnosis #14, sensor #3
32403919	Chen K et al., 2020	diagnosis #16, imaging #6
39930702	Liu X et al., 2025 (PET)	diagnosis #19, imaging #9
33541073	Chen J et al., 2021	diagnosis #21, imaging #10
36082964	Xu Z et al., 2022	diagnosis #24, imaging #8
39835521	Chen L et al., 2025	diagnosis #28, sensor #6
26190471	Mir TA et al., 2015	diagnosis #31, sensor #5
26763166	Zhao L et al., 2016	diagnosis #33, capture #2
30977684	Shende P et al., 2019	diagnosis #35, liquid_biopsy #3
34159726	Xu M et al., 2021	diagnosis #46, imaging #15
26200911	Wu C et al., 2015	diagnosis #48, imaging #16
37783108	Lv W et al., 2024	diagnosis #51, capture #9
38270088	Wang M et al., 2024	diagnosis #55, imaging #19
30565898	Dong Z et al., 2018	diagnosis #61, capture #8
23682757	Zhang C et al., 2013	diagnosis #62, imaging #20
33724284	Li J et al., 2021	imaging #11, capture #5
39157104	Xu X et al., 2024 (CD63)	sensor #4, capture #3
